# Differentiating acute from chronic insomnia with machine learning from actigraphy time series data

**DOI:** 10.3389/fnetp.2022.1036832

**Published:** 2022-11-28

**Authors:** S. Rani, S. Shelyag, C. Karmakar, Ye Zhu, R. Fossion, J. G. Ellis, S. P. A. Drummond, M. Angelova

**Affiliations:** ^1^ School of Information Technology, Deakin University, Geelong, VIC, Australia; ^2^ Centro de Ciencias de la Complejidad (C3) and Instituto de Ciencias Nucleares, Universidad Nacional Autónoma de México, CDMX, Mexico; ^3^ Instituto de Ciencias Nucleares, Universidad Nacional Autónoma de México, CDMX, Mexico; ^4^ Department of Psychology, Northumbria University, Newcastle Upon Tyne, United Kingdom; ^5^ Turner Institute for Brain and Mental Health, School of Psychological Sciences, Monash University, Melbourne, VIC, Australia

**Keywords:** acute insomnia, chronic insomnia, actigraphy, machine learning, insomnia detection, dynamical features, sleep parameters

## Abstract

Acute and chronic insomnia have different causes and may require different treatments. They are investigated with multi-night nocturnal actigraphy data from two sleep studies. Two different wrist-worn actigraphy devices were used to measure physical activities. This required data pre-processing and transformations to smooth the differences between devices. Statistical, power spectrum, fractal and entropy analyses were used to derive features from the actigraphy data. Sleep parameters were also extracted from the signals. The features were then submitted to four machine learning algorithms. The best performing model was able to distinguish acute from chronic insomnia with an accuracy of 81%. The algorithms were then used to evaluate the acute and chronic groups compared to healthy sleepers. The differences between acute insomnia and healthy sleep were more prominent than between chronic insomnia and healthy sleep. This may be associated with the adaptation of the physiology to prolonged periods of disturbed sleep for individuals with chronic insomnia. The new model is a powerful addition to our suite of machine learning models aiming to pre-screen insomnia at home with wearable devices.

## 1 Introduction

Reduced or disturbed sleep is increasingly recognised as presenting a significant health risk, and has been associated with increases in a diverse range of health-related problems and reduced quality of life (Wickwire et al., 2019). In terms of quality and quantity, sleep is affected by various sleep disturbances of which insomnia is one of the most common. According to the Diagnostic and Statistical Manual of Mental Disorders, fifth edition (DSM-5), insomnia is defined as dissatisfaction with the quality or quantity of sleep which can include difficulty falling asleep, difficulty maintaining sleep (with frequent awakenings or problems returning to sleep after awakening) and/or waking up early and being unable to get back to sleep and the resultant daytime impairments (Edition et al., 2013). Depending on its duration, insomnia can be classified into acute or chronic. Acute and chronic insomnia have different underlying causes and may require different treatments.

Acute insomnia (AI) is a brief episode of difficulty sleeping, which may last from several nights up to 3 months. It can be caused by a significant life stress, illness, effects of some medications or drugs, a pandemic, emotional or physical discomfort, environmental discomfort, major job or relationship change (Ellis et al., 2012, 2014; Altena et al., 2020). Chronic insomnia (CI) is a longer-term pattern when a person has difficulty sleeping for at least 3 nights a week for 3 months or longer. CI has many potential causes including chronic stress, depression or anxiety, pain or discomfort at night. CI has been associated with daytime cognitive deficits, exhaustion, a range of psychiatric and physical morbidities, reduced quality of life, as well as increased use of health services (Wilkerson et al., 2012; Taylor et al., 2013). AI has been associated with the first onset of depression (Ellis et al., 2014). Furthermore, if untreated, it can convert to CI with transition rates of 21.43% for large population samples in the US and the United Kingdom (Ellis et al., 2012).

Differentiating AI from CI and early diagnosis is essential for successful treatment. While insomnia’s prevalence and incidence are high, the condition is under-diagnosed and often untreated (Williams et al., 2018; Morin et al., 2020). Thus, a considerable number of individuals with insomnia do not seek medical attention, and may not even realise that their sleep is unhealthy.

The clinical assessment of AI and CI is usually based on self-reported symptoms from the individuals and their sleep diaries (Riemann et al., 2017). However, sleep diaries are subjective and can be burdensome for the individual. The clinical assessment could be complemented by polysomnography (PSG) and actigraphy. PSG is usually performed in a sleep laboratory. Its primary use is not to assess AI and CI but to rule out other sleep disorders, such as sleep apnoea or restless leg syndrome. As individuals with insomnia usually demonstrate night-to-night variability in their sleep, one or two nights of PSG are unlikely to be representative (Buysse et al., 2010). Besides, these individuals often sleep well in the laboratory outside their home environment (Baglioni et al., 2014).

Actigraphy provides cheap and non-invasive means for continuous monitoring of human rest/activity patterns and is increasingly used for sleep monitoring over long periods of time. Actigraphic devices can infer sleep characteristics from the physical activity (Nakazaki et al., 2014) and are widely used to measure sleep in a home environment over several nights. This is particularly suitable for monitoring AI and CI as it can monitor sleep in a natural home environment over multiple nights (Angelova et al., 2020a; Hamill et al., 2020; Walters et al., 2020; Kusmakar et al., 2021).

In a series of papers (Holloway et al., 2014; Fossion et al., 2017; Angelova et al., 2020a; Kusmakar et al., 2021), several markers were investigated for AI and CI using 7 nights of continuous actigraphy monitoring. Automatic models were proposed for the classification of acute insomnia from normal sleep (Angelova et al., 2020a) and chronic insomnia individuals from their bed partners (Kusmakar et al., 2021) without the need of sleep diaries.

Fractal analysis techniques were implemented for the first time (Holloway et al., 2014) to study acute insomnia using actigraphic data. Power spectrum and detrended fluctuation analysis (DFA) were used to search for 1/*f* scaling, meaning that the power in the signal is dominated by an inverse power law with the frequency *f*. 1/*f* scaling is associated with a long-range correlation of the time series and high complexity (Wagenmakers et al., 2004), measured by the complexity parameter ∼1 . It was concluded that the variations in 1/*f* − type of scaling in the nocturnal signal of individuals with acute insomnia compared to healthy controls is in the range 0.75–1.25 corresponding to long-range correlations in the time series, owing to increased night-time arousals. The healthy controls displayed a complexity parameter in the range of 0.5–0.75, associated with positive but weaker correlations in the time series. The effect of circadian rhythms was also investigated on the population of individuals with acute insomnia compared to healthy controls using complete day-night actigraphy (Fossion et al., 2017). A later study indicated that a hyper-vigilance state in people with insomnia may indicate an increased risk of cardiovascular disease (Laharnar et al., 2020). The works (Angelova et al., 2020a; Kusmakar et al., 2021) proposed automatic models to distinguish acute insomnia from normal sleep and chronic insomnia from bed partners using actigraphy data. The models were based on a 2-layer machine learning algorithm, where the first layer is the classifier and the second layer is the optimisation. In order to distinguish insomnia from a healthy sleep, the models first predicted the quality of each night of sleep for each individual, followed by the classification of the individuals to insomnia or normal sleep type. The model differentiated AI from healthy sleep with accuracy 80%, sensitivity 76%, and specificity 92%. A second model was developed to distinguish between CI and their bed partners (Kusmakar et al., 2021).

Motivated by the differences between AI and CI and the success of our models to classify insomnia from a healthy sleep, in this paper, we go a step further and develop a robust model to distinguish AI from CI. We have combined the data from the two sleep studies together. Taking into account the limitations and challenges of the data, we hypothesise that:

Hypothesis 1: Our model can distinguish acute insomnia from chronic insomnia.

Ultimately, we propose a new automatic model to differentiate between AI and CI. Furthermore, we demonstrate the observed changes in CI patterns of actigraphy recordings are smaller compared to patterns for individuals with AI and more similar to bed partners and healthy controls. We speculate that:

Hypothesis 2: Observed changes in patterns of CI and AI individuals may appear because the homeostatic drive has adjusted to sleeplessness in the individuals with chronic insomnia, while for those with acute, the changes resultant from the acute insomnia are still too raw and the organism and the respective homeostatic regulation have not adapted to these changes yet.

The paper is organised as follows. Section 2 describes the data, descriptive statistics, and methods for the feature extraction and the design of the machine learning model. The results and analysis are given in Section 3 and discussed in Section 4, where our hypotheses are verified, followed by our conclusions in Section 5.

## 2 Methods

### 2.1 Data

Our analysis of individuals with acute insomnia (AI) employs actigraphy time series data from publicly available data sets (Holloway et al., 2014). The data collected for the original study was approved by the University of Glasgow Ethics Committee. The chronic insomnia (CI) data set was obtained from a larger clinical trial (Project REST; Australian New Zealand Clinical Trials Registry Registration: ACTRN12616000586415) and approved by Monash University’s human research ethics committee (Mellor et al., 2019) to investigate behavioural interference because of sleep partner (Mellor et al., 2019; Walters et al., 2020). The subset of data from the individuals with chronic insomnia and their bed co-inhabiting partners used here is publicly available (Angelova et al., 2020b).

Often, older adults with age > 60 years have an earlier bedtime and wake-up time, as their circadian rhythm is advanced. Sleep architecture changes include spending a greater proportion of time in different stages of sleep, indicating a reduction in deep, rejuvenating sleep and an elevation in superficial and transient sleep (Rodriguez et al., 2015). Moreover, older adults tend to sleep for a shorter period of time than their younger counterparts. In order to exclude the effects of ageing on normal sleep and insomnia, we included only data from adults with ages ≤60 years in our analysis.

The first data set has 49 adults (age: 18–60 years) including the 22 asymptomatic healthy controls (HC) (average age: 27.82 ± 5.55 years) and 27 individuals with AI (average age: 30.74 ± 11.16 years). The second data set was collected from the group of 65 adults (age: 18–60 years) including 32 adults suffering from CI (average age: 43.06 ± 11.81 years) and their respective bed partners (BP) (average age: 42.73 ± 12.55 years). These two groups were age matched because of the partner status. Here, we are mainly interested in the AI and CI groups, but also provide descriptions for the healthy groups so comparisons can be made where possible.

In the AI data set, the control cohort was composed of self-declared healthy subjects with no known problem with sleeping, and the insomnia cohort (clinically assessed) had no known co-morbidity. All subjects were requested to remain in bed between 10 pm and 8 am next day. However, this did not prohibit the subject from going to bed before 10 pm or leaving the bed after 8 am. An actiwatch device (AW4, Cambridge Neurotechnology, pre-2014) was used to collect data from adults with acute insomnia and healthy sleepers. Data were collected for 2 weeks, however not all subjects completed the entire 2-week period. The majority of the individuals were young adults (age: 18–40 years) for which the data were completed for 1 week. In addition, the actiwatch lacked the functionality to detect lights out, which made it difficult to calculate accurately from the signal two traditional sleep parameters, namely sleep latency and sleep efficiency, from the AI data set. Respironics Actiwatch Spectrum Pro and Actiware software (Respironics, Bend, OR, United States) were used to collect and pre-process the CI data set. This device had the functionality to detect lights out. Individuals with CI and their co-inhabiting bed partners wore the devices at all times for 1 week.

**FIGURE 1:**
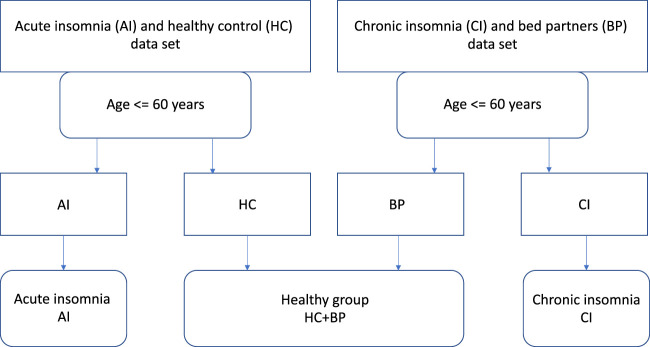
Data inclusion/exclusion flowchart.

For this paper, we combined the two data sets and integrated activity counts over 1 min epochs for seven nights of actigraphy data. The total number of subjects, male and female numbers, mean age with standard deviation (s.d.) and total number of nights recording used to build the model are given in Table 1. The data inclusion/exclusion flowchart is shown in Figure 1.

**TABLE 1:** Total number of subjects, male and female, average age and its standard deviation (sd) and total number of nights of actigraphy recordings of the subjects in the two studies used in the model.

	Dataset 1		Dataset 2	
Subjects	AI	HC	CI	BP
# Subjects	27	22	32	33
Gender	Male–5	Male–8	Male–8	Male–22
	Female–18	Female–14	Female–24	Female–11
	Unknown-4			
Mean age	30.74	27.82	43.06	42.73
s.d. (Age)	11.16	5.55	11.81	12.55
# Nights	189	154	224	231

### 2.2 Data pre-processing

Night-actigraphy data (from 10 pm to 8 am) are used for the analysis presented in this paper, as the focus is on the night activities only. This also excludes noisy unlabelled daily activities the individuals were involved in, and subjective bias, as the data for the studies were collected in an uncontrolled environment. Missing data (approximately 1%) in each actigraphy time series were handled using the moving median method with the sliding window of 30 min. The logarithmic transformation (log_2_) was applied on the actigraphy time series for further analysis to reduce the effect of skewed distribution on feature extraction. In addition, since two different devices from two different vendors were used to record the actigraphy data, the logarithmic transformation also helped to bring the recordings in a similar range.

**FIGURE 2:**
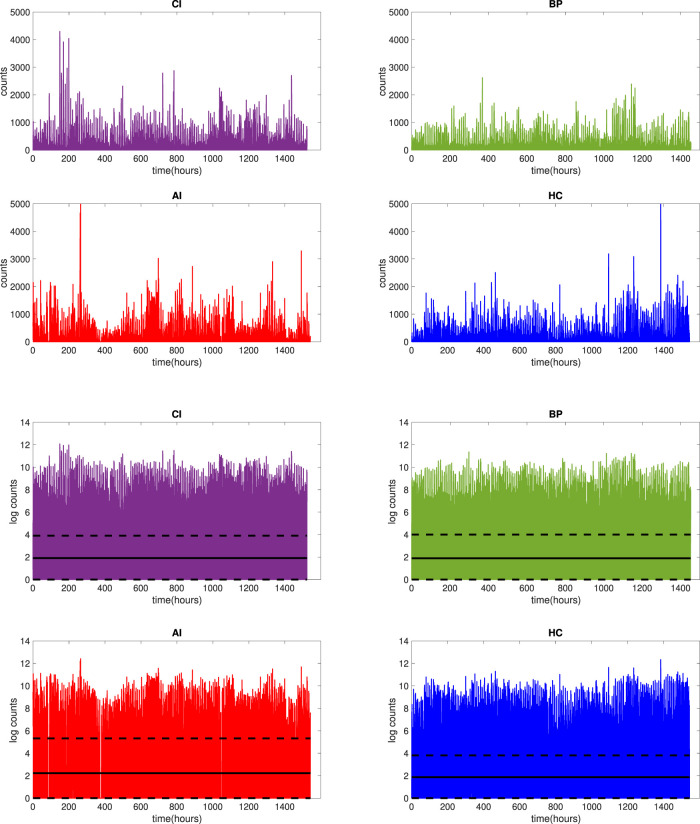
Raw actigraphy signals (top 4 panels) and log_2_-transformed actigraphy signals (bottom 4 panels) for 7 nights of actigraphy for 22 participants in each group: CI (purple), BP (green), AI (red) and HC (blue). The solid black line (bottom 4 panels) represents the mean of the signal, while dashed lines represent 25 and 75 percentiles of the signal.

Figure 2 presents the raw and log_2_-transformed nocturnal actigraphy signal for 22 individuals (the minimal number of subjects available per group) in each group CI (purple), BP (green), AI (red) and HC (blue) respectively. So the length of the horizontal axis, presenting the length of the data in hours for 7 nights of recording from 22 subjects for 10 h per night, is 22 × 7 × 10 = 1,540 h. The solid black line plotted in Figure 2 depicts the mean, while the dashed lines determine the 25th and 75th percentiles of the plotted data. From visual inspection of the plot across 22 subjects, it is obvious that the log-transformed activity counts showed the same range for each group CI (adults with chronic insomnia), BP (bed partners of CI), AI (adults with acute insomnia) and HC (healthy control). Therefore, the study design is not affected by the measurement devices and for the purpose of this work, we merged BP and HC groups into one healthy group (HC + BP). In the remaining of this paper, we will use HC + BP for presenting this combined healthy control group.

### 2.3 Design of experiment

**FIGURE 3:**
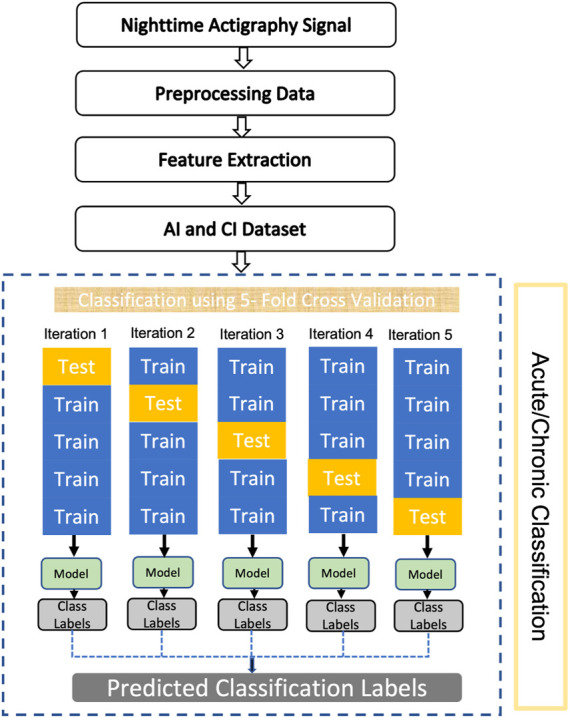
Workflow of the proposed model for AI and CI classification.

The experiment was designed to include the following steps in the data analysis cycle: input of the raw data, pre-processing of data, feature extraction, machine learning model and classification. The workflow of the proposed model for the AI and CI classification is shown in Figure 3. Similar models were designed for other pairs of groups: AI and HC + BP, CI and HC + BP, insomnia (AI and CI) v/s HC + BP.

### 2.4 Feature extraction

To analyse the night actigraphy we extracted statistical and dynamical features from the night time signals. The following features were calculated: arithmetic mean, standard deviation, complementary cumulative distribution, intradaily variability and stability, complexity parameter *α* calculated using DFA, power law index *β* from power law, Higuchi fractal dimension (HFD) obtained from Higuchi algorithm, and Shannon entropy (ShE). Furthermore, three sleep parameters were used, total sleep time (TST), wake after sleep onset (WASO), and sleep-wake ratio (SWR), which can be extracted from all signals.

Since there is no specific threshold for the amplitude of the actigraphy signal for wake detection (Natale et al., 2014), we have used an amplitude equal to zero to identify sleep and an amplitude threshold greater than zero to identify wake.

*Statistical Analysis:* Statistical analysis is performed to determine the overall view of both data sets. Mean and standard deviation are calculated from the transformed data to ascertain the average variance and magnitude of the night time signal of adults suffering from chronic insomnia and their respective bed partners as well as people with acute insomnia and same age healthy controls.

*Intradaily Variability* (IV) calculation is non-parametric and gained popularity in physiological and actigraphic time series analysis (Witting et al., 1990; Gonçalves et al., 2015). IV is applied to time series *X*_*i*_, *i* = 1,... *N*, sampled at 60-min intervals, and defined as:

$$IV=\frac{N\sum_{i=2}^{N}{\left(X_{i}-X_{i-1}\right)}^{2}}{\left(N-1\right)\sum_{i=1}^{N}{\left(X_{i}-\bar{X}\right)}^{2}},$$ (1)

where $\bar{X}$ is the arithmetic mean taken over all data *X*. IV provides the significant information about the amount of variance present in time series and can be used to demonstrate the variation in the activity patterns.

*Intradaily Stability* (IS) is the estimation of how steady the rhythm of the time series is over several nights. It shows the similarity in the night patterns. IS is calculated as the proportion of the difference of the average activity pattern to the overall variation, according to

$$IS=\frac{N\sum_{i=1}^{P}{\left(\bar{X_{i}}-\bar{X}\right)}^{2}}{P\sum_{i=1}^{N}{\left(X_{i}-\bar{X}\right)}^{2}},$$ (2)

where *N* is the total number of data points or values, *P* is the number of hourly means per day, $\bar{X}$ is the mean value for all data *X*, $\bar{X_{i}}$ is the hourly mean value for the data *X*, *X*_*i*_ is the individual data point, *i* = 1, …*N*. IS varies between 0 and 1. 0 indicates Gaussian noise. Smaller values of IS indicate higher variation in signal (Witting et al., 1990; Blume et al., 2016).

*Complementary cumulative distribution function* (CCDF): represents the complementary collective distribution feature, a statistical power estimation technique that can just be performed on time-domain data. It estimates if the probability *P* of power of the given signal *F* will be above the specified average signal power level *x*. CCDF can be written as

$${\bar{F}}_{X}\left(x\right)=P\left(X>x\right)=1-F_{X}\left(x\right).$$ (3)

CCDF accentuates the peak or maximum values as it provides the probability of signal power to be above the certain value. The CCDF highlights power levels at their maximum or peak. The CCDF is regarded as one of the most significant statistical measurements, and it is employed in a broad range of applications.

*Power Spectrum Analysis* (PSA) is the classical approach to investigate the properties or features of any signal (Guzman-Vargas et al., 2011). Its primary objective is to show the dominant frequencies in order to demonstrate periodicities in the data. PSA also investigates the existing self-affinity or correlations in real-time signal or time-series. A power spectrum is derived from the data using Fourier transform, and then the following dependence is sought:

$$S\left(f\right)\propto f^{-\beta },$$ (4)

where *S*(*f*) is the power, *f* is the frequency and *β* is scaling parameter. *β* is calculated from the slope of the graph of the logarithms of *S* and *f*. The slope obtained from the graph provides an insight of any self-similarity present in the signal.

The value range for *β* between 0 and 2 is of interest for physiological and physical motion data, where 0 indicates no correlation (white noise), 1 represents long-range correlation, known also as 1/*f* noise or pink noise, and 2 indicates short-range correlation or random walk (Brownian noise) (Guzman-Vargas et al., 2011). The relations between the scaling parameters and correlations in the signal are given in Table 2.

**TABLE 2:** Relations between correlations and scaling parameters.

Description	*α*	*β*	HFD
Uncorrelated, white noise	∼0.5	∼0	∼2.5
Positively correlated	>0.5	>0	<2.5
1/f noise, pink noise	∼1	∼1	∼2
Brownian noise, random walk	∼1.5	∼2	∼1.5

*Detrended Fluctuation Analysis* (DFA) is an effective tool to study statistically the scale of auto-correlation and self-affinity in a varying signal (Peng et al., 1994). DFA removes the external stimulated local trends in order to investigate the irregular correlations (Seely and Macklem, 2004). The method is based on splitting the time series into shorter parts (boxes) of the same size and fitting the least squares line for each short time series. DFA calculates the signal’s mean squared distance from its local trend line to get a scaling parameter *α*. The complexity of a signal in DFA is analysed using the series *F*, defined as follows:

$$F\left(n\right)=\sqrt{\frac{1}{n}\overset{N}{\underset{k=1}{\sum }}{\left[y\left(k\right)-y_{n}\left(k\right)\right]}^{2}},$$ (5)

where *N* is the length of the time-series, *n* is the size of the box, *y*(*k*) is the integrated time-series and *y*_*n*_(*k*) represents the local trend. Each box is then subjected to the same process to establish a relationship between average local fluctuations. Log-log plots are then used to determine if there is a linear relationship, indicating whether self-similar scaling exists, which is denoted by the parameter *α*.

The scaling parameter *β*, determined from the power spectrum, and *α*, calculated with DFA, are both used to reveal the correlation in time series (Peng et al., 1995). Notably, the theoretical parameters are linearly dependent: *β* = 2*α*–1, (Table 2).

*Higuchi Fractal Dimension* (HFD): Mandelbrot introduced the term Fractal Dimension (FD) (Mandelbrot et al., 1983) to describe fractals, which represent self-similar, infinite and complex pattern objects. FD can be obtained by measuring the changes in the scaling and is also used to investigate the complexity of a signal. Among all the available algorithms to calculate FD, Higuchi algorithm provided most accurate results (Raghavendra et al., 2009). According to Higuchi algorithm, from a time series taken at regular intervals, *x*(1), *x*(2), *x*(3),……, *x*(*N*), a new time series is constructed, $x_{n}^{k}$ , by splitting into equal *k* time series, defined as follows,

$$x_{k}^{n}=\left\{x\left(n\right),x\left(n+k\right),x\left(n+2k\right),\ldots ,x\left(n+\left\lfloor \frac{N-k}{k}\right\rfloor \right)\right\}.$$ (6)

Here, *n* = 1, 2, 3, ….*k* (*n* is the initial time and *k* is the interval length) and $\left\lfloor .\right\rfloor$ is an integer function representing the nearest lower integer value for a real number. The length of the curve *L* for each *k* time series is given as

$$L_{n}\left(k\right)=\frac{1}{k^{2}}\left(\begin{array}{c} \overset{\left\lfloor \frac{N-n}{k}\right\rfloor }{\underset{i=1}{\sum }}\left(x\left(n+ik\right)-x\left(n+\left(i-1\right)k\right)\right) \end{array}\right)\left(\begin{array}{c} \frac{N-1}{\left\lfloor \frac{N-n}{k}\right\rfloor } \end{array}\right)$$ (7)

where *N* is the length of the full time series, and $\left\lfloor \frac{N-n}{k}\right\rfloor$ is normalisation factor. The fragments *L*_*n*_(*k*) are summed to give the length of the fractal curve *L*(*k*). The value of fractal dimension *D* is estimated as the slope of the best linear fit to the calculated data points $\left\{\left(\log\frac{1}{k},\log L\left(k\right)\right)\right\}$ .

The calculated value *D* = 1 means a simple curve while *D* = 2 represents a plane (Klonowski et al., 2004). In one dimension, for theoretical fractal motion, the relationship between *β* and FD, *D*, for 1 < *β* ≤ 3 is given by (Cervantes-De la Torre et al., 2013):

$$D=\frac{5-\beta }{2}=3-\alpha .$$ (8)

Thus, for white noise, *β* ∼ 0, *D* ∼ 2.5, for pink noise (1/*f* similarity), *β* ∼ 1, *D* ∼ 2, while for short range correlations (Brownian noise) *β* ∼ 2, *D* ∼ 1.5 (Table 2). Real physical motion time series often deviate from the theoretical fractals and the relation given by Eq. 8 is used mainly for guidance.

*Shannon Entropy* (ShE): Shannon introduced the idea of information entropy to measure the amount of information transmitted by a message or contained in a signal (Shannon, 1948).

According to Shannon’s method any random variable *x*(*n*) can contain *N* possible values, and the probabilities of these values are *p*_1_, *p*_2_,…, *p*_*N*_. ShE is defined according to:

$$ShE=-\frac{1}{\log N}\overset{N}{\underset{i}{\sum }}p_{i}\log p_{i},$$ (9)

where *N* is the length of the message or a signal, or total number of events/values, and *p*_*i*_ is the probability of the *i*th event/value.

*Acigraphy-derived Sleep Parameters:* The sleep parameters TST, WASO and SWR were calculated from the night time actigraphy signal (Nakazaki et al., 2014) for both datasets. SWR, sleep-wake ratio (Nakazaki et al., 2014; Kusmakar et al., 2021) is calculated by following equation:

$$SWR=\frac{TST}{WASO}.$$ (10)

For both datasets, we have considered data from 10 pm to 8 am for seven nights. For the CI dataset, where a modern actiwatch was used, the proprietary software of the watch determined when the participants went to sleep and ended their sleep automatically. Time in bed (TIB) was calculated based on rest intervals generated by the device, WASO—as the sum of all wake epochs (activity count is non-zero) between sleep onset (SO) time and sleep end, TST—as the sum of all sleep epochs (activity count is zero) between SO and sleep end. For the AI dataset, WASO was calculated with start (10 pm) and the end of the sleep cycle (8 am) and TIB was empirically selected as 10 h. SWR was calculated from Eq. 10 for both datasets.

### 2.5 Machine learning model

This study proposes a classification model to differentiate individuals with AI from individuals with CI using the features extracted from the actigraphy data as described in the previous sections. The data were stratified by gender in the training and testing sets to make sure that gender covariates were appropriately adjusted.

We labelled AI and CI individuals in the response variable as -1 and 1 for the modelling purpose. The labelled datasets were divided into training and testing datasets using *k*-fold cross-validation approach. In this process, the model is trained *k* times using (*k* − 1) folds of data and used the remaining fold as a test set. In this study, we used *k* = 5 for this cross-validation. We applied min-max normalisation on the training dataset and recorded the minimum and maximum values, which were then used for normalising the corresponding test dataset. At each iteration, model hyperparameters were optimised using the auto-optimisation option provided in Matlab. This option uses training samples only and finds hyperparameters that minimise five-fold cross-validation loss. Then the performance on the test dataset was recorded using the optimised model. To eliminate the effect of random sampling, the entire process was repeated five times. Finally, the averaged performance was reported for each machine learning model. Figure 3 illustrates the five-fold cross-validation process. Four machine learning models were built based on *k*-nearest neighbour (kNN), support vector machine (SVM), Naïve Bayes (NB) and Random Forest (RF) algorithms. The performance of the models was evaluated using the testing data. Due to the small amount of data, not sufficient for deep learning algorithms to perform well, and the need for explainability of our models, only traditional machine learning algorithms were selected.

1) *k*-Nearest Neighbour (kNN) is widely used non-parametric method for classification and regression analysis (Fix and Hodges, 1951). Given a labelled data set and a new unlabelled datum, it assigns a label to the datum in accordance to the majority label among *k* nearest neighbouring data points to the datum. In other words, it assigns new data values based on how closely they match the values in the training sets. For the purpose of training the model, a distance metric is calculated between *k* nearest neighbors. The data is then classified according to the nearest neighbour. The trained model can then be applied to classify new data.
2) Support Vector Machine (SVM) is a popular supervised machine learning method, used here for classification of AI and CI. Its aim is to increase separation of different clusters in the data by projecting it into a higher-dimensional hyperspace, therefore, simplifying linear classification by separating hyperplanes (Cortes and Vapnik, 1995).
3) Naïve Bayes (NB) is an efficient classification algorithm for supervised learning based on the Bayes theorem with the assumption that the presence of a particular feature in a class is “naively” completely independent (unrelated) of any other feature (Nisbet et al., 2009). NB algorithm calculates the output based on the conditional probabilities of the data.
4) Random Forest (RF) is another prominent machine learning classification algorithm. This method works on the construction of large sets of decision trees by using randomly selected features from the training data sets with bootstrap or bagging aggregation (Breiman, 2001). RF method generates results by computing the mean of the outcomes from the decision tree. RF model increases the accuracy of the model by reducing the overfitting of data.

### 2.6 Performance metrics

After constructing the classification models and calculating the results, sensitivity, specificity and accuracy are procured as a standard method (Powers, 2020) to assess the efficiency of selected classifiers. These standard measures are determined by comparing the predicted classes with the ground truth and calculating true positives (*TP*), true negatives (*TN*), false negatives (*FN*) and false positives (*FP*),

1) Accuracy $=\frac{TP+TN}{TP+TN+FP+FN}\times 100$ , is the percentage of correctly detected classes;
2) Sensitivity $=\frac{TP}{TP+FN}\times 100$ , is the percentage of correctly predicted positive values;
3) Specificity $=\frac{TN}{TN+FP}\times 100$ , is the percentage of correctly predicted negative values;
4) F-score $=2\frac{Precision\times Recall}{Precision+Recall}\times 100$ . It represents a harmonic average of the recall (sensitivity) and precision, where the precision is the ratio of correctly identified classes (including true and false positives) to all correct classes.

As the first task is to classify acute *versus* chronic insomnia, acute insomnia is labelled as “True” and chronic insomnia is labelled as “False”. Then, *Sensitivity* extracts the percentage of correctly detected individuals with AI while *Specificity* identified the percentage of correctly detected individuals with CI. *Accuracy* provides the percentage of correct detection of individuals with AI and CI.

AUC (area under the curve) is another important measure to evaluate the performance of machine learning model. The value of AUC represents how well a model is capable of categorising between the classes (Huang and Ling, 2005).

*Mann-Whitney U test* is a non-parametric test to analyse the mean of given variable and check whether the distribution of given data is different or similar (Ruxton, 2006). The Mann-Whitney *U* test is performed by ranking the data for each condition and then comparing how different the two ranks are. Consequently, when the two conditions are different, then most high-ranking data will belong to one of the conditions and most low-ranking to the other. Thus, the ranks will differ considerably. However, if both conditions are similar, the low and high ranks will be allocated fairly equally between the two conditions, which will result in a similar rank totals.

## 3 Results

In this paper we have used seven nights of nocturnal actigraphy data to extract the features using the methods described in Section 2. The data were log-transformed to take into account that the data collections for the two sleep studies, acute and healthy individuals, and chronic insomnia individuals and their partners, were done with two different devices. The average values of the seven nights are used for training and testing the machine learning model.

We have also transformed the values of all features using the min-max transformation to (0,1) range in order to construct machine learning models for automated detection and differentiation of adults with AI from adults with CI.

The arithmetic means ± standard deviations of all calculated features for 7 nights of actigraphy of AI, CI, and HC subjects of age ≤60 years are given in Table 3.

**TABLE 3:** Mean ± sd of (unscaled) features for individuals of age between 18 and 60 years old with AI, CI, and HC + BP. The statistical and dynamical features are calculated using log transformed signal data. The actigraphy-derived sleep parameters TST and WASO are given in minutes, while SWR is dimensionless. Four AUC values are given—AUC_1_ (AI vs. CI), AUC_2_ (AI vs. HC + BP), AUC_3_ (CI vs. HC + BP) and AUC_4_ (Insomnia vs. HC + BP). The * indicates that a negation is used for the AUC value <0.5 . *p*-value (AI vs. CI) is calculated using Mann-Whitney *U* test.

Feature	CI	AI	HC + BP	*p*	AUC_1_	AUC_2_	AUC_3_	AUC_4_
Statistical features								
Mean	1.90 ± 0.42	2.14 ± 1.00	1.87 ± 0.51	0.66	0.53	0.55	0.54	0.54*
Sd	3.04 ± 0.31	2.89 ± 0.42	2.90 ± 0.29	0.23	0.59*	0.50	0.63	0.57*
IV	0.74 ± 0.18	0.80 ± 0.21	0.79 ± 0.15	0.37	0.57	0.51	0.57*	0.53
IS	0.14 ± 0.05	0.16 ± 0.05	0.14 ± 0.04	0.04	0.66	0.61	0.58*	0.51*
CCDF	0.56 ± 0.02	0.57 ± 0.03	0.56 ± 0.02	0.24	0.59	0.56	0.56*	0.51
Dynamical features								
*β*	0.66 ± 0.12	0.68 ± 0.12	0.63 ± 0.11	0.56	0.55*	0.64*	0.59	0.61
*α*	0.86 ± 0.11	1.01 ± 0.09	0.90 ± 0.13	1.8E-06	0.86	0.76	0.60*	0.57*
HFD	1.85 ± 0.03	1.88 ± 0.04	1.87 ± 0.03	3.4E-04	0.77	0.61	0.68*	0.55
ShE	2.34 ± 0.38	2.44 ± 0.78	2.35 ± 0.43	0.80	0.52*	0.51*	0.52*	0.51
Sleep parameters								
TST	381 ± 42	367 ± 92	388 ± 53	0.65	0.54	0.52*	0.56*	0.54
WASO	82 ± 20	87 ± 86	75 ± 27	0.03	0.67*	0.58*	0.64	0.54*
SWR	5.7 ± 2.8	9.0 ± 4.8	6.7 ± 3.1	6.7E-04	0.76	0.67	0.65*	0.50

### 3.1 Feature values

#### 3.1.1 Statistical features

The mean values of the amplitude of physical activity for AI subjects is higher compared to the means of CI and HC + BP. This indicates that individuals with AI have higher physical activity than those with CI, which supports our Hypothesis H2. Furthermore, the mean of the healthy sleepers, while smaller than the mean of AI cohort, is similar to the mean of the CI cohort, which supports partly our Hypothesis H1. The standard deviations (sd) for all three groups are very similar.

The intradaily variability, IV, of the AI population is larger compared to the CI and HC + BP group. High value of IV stipulates more waking up, and more physical activities, during nighttime (Witting et al., 1990).

The intradaily stability, IS, is very similar for the CI and HC + BP groups and smaller than the AI group. The results for the complementary cumulative distribution function, CCDF, indicate slightly larger values for the AI group compared to the other two groups, for which the values are similar. This partly supports our Hypothesis H2.

#### 3.1.2 Dynamical features

The parameter *β*, computed from the power spectrum, is 0.68 for AI and 0.66 for CI, showing that these two populations have similar complexity patterns with slightly higher complexity for the AI group. The values of *β* for all three populations are in the range of the 1/*f* noise and indicate the presence of long-range correlations in the time series.

**FIGURE 4:**
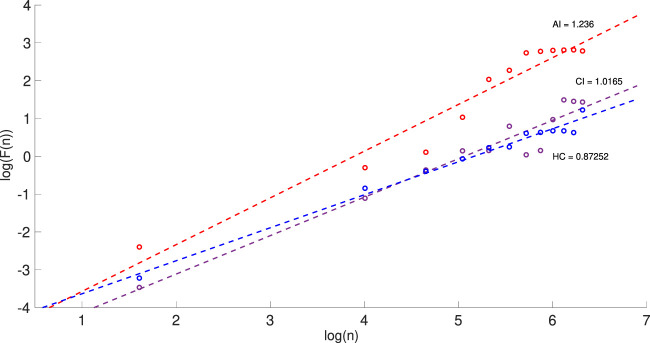
Detrended fluctuation analysis (DFA) for one night of the actigraphy showing the log-log plots of *F*(*n*) against the box size *n* for three individuals with AI (red), CI (purple) and HC (blue). The slope of each line determines the values of *α* for each individual. The range for *n* is from 5 to 600 with box selection of 50 in each plot.

The average value of the complexity parameter *α* shows that the physical activities for the AI group are more complex and thus must be also more nocturnal activities, compared to the CI group. This supports our Hypothesis H2. Figure 4 illustrates the results gained from DFA for one night of actigraphy from one individual per group of a similar age.

The Higuchi fractal dimension, HFD, shows an average value for the AI subjects of 1.88 compared to 1.85 for the CI subjects. It indicates 1/*f* behaviour (Table 2) and, as expected, is in agreement with the results obtained from PSA and DFA for *β* and *α* respectively. The higher value of HFD for AI is possibly due to more nocturnal awakenings of this group compared to CI and HC + BP. This partly supports the Hypothesis H2.

The Shannon entropy, ShE, shows a slightly higher average value in AI cohort in comparison to CI and HC + BP groups which may indicate more night time disturbance.

**FIGURE 5:**
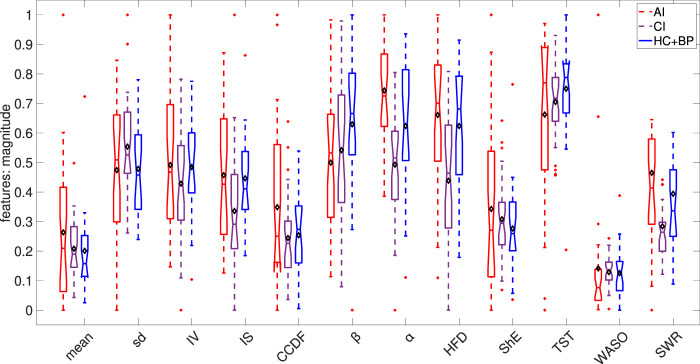
Box plots of the calculated statistical, dynamical and sleep features. Each feature was rescaled to the range of (0,1) based on the minimum and maximum over the combined AI, CI and HC + BP cohorts. In each box plot, the diamond shape indicates the mean and the horizontal line depicts the median of the rescaled feature.

Figure 5 shows the box plots of the statistical, dynamical and sleep features of the studied signals. It illustrates that the night time signal for the AI cohort has more variation, as demonstrated by the statistical features, suggesting more night time physical activity in AI subjects in comparison to those suffering from CI and further supports our Hypothesis H2.

#### 3.1.3 Sleep parameters (features)

Three sleep parameters were calculated for all three groups (AI, CI, and HC + BP). The results in Table 3 show that the average TST in minutes for the AI cohort is less than that for the CI cohort, which in turn is less than the one for HC + BP.

The average wake after sleep onset, WASO, is highest for the AI group, followed by the WASO for CI and HC + BP, with a consistent pattern of larger WASO for the insomnia sufferers compared to healthy sleepers.

The sleep wake ratio, SWR, was calculated for all individuals for 7 nights of actigraphy and the average results are given in Table 3. While the SWR for the AI group is almost twice as large compared to the one for the CI group, this may be due to the requirement for staying in bed, while they are not actually sleeping, imposed in one of the studies. We note the relatively larger standard deviation for the AI group for all three sleep parameters, which may be a result of the same protocol requirement.

The *p*-values, computed with Mann-Whitney *U* test, are given in Table 3. *p* < 0.05 indicates that the feature is statistically significant to classify the difference between individuals with acute and chronic insomnia. Thus, *α*, HFD, IS, WASO and SWR are highly statistically significant to differentiate between the two groups.

The calculated AUC values of the features are given in the last columns of Table 3: AUC_1_ is for AI vs. CI, AUC_2_ for AI vs. HC + BP, AUC_3_ for CI vs. HC + BP and AUC_4_ for Insomnia (AI and CI) vs. HC + BP. Negation is applied to the features with AUC values <0.5 (to report the discriminative power of the feature in the 0.5-1 range) and the corresponding values are indicated with * in the Table.

Regarding AI and CI groups, only two features, *α* and HFD, have relatively high values of AUC_1_ of 0.86 and 0.77 respectively, to be used as single markers capable to differentiate between the AI and CI groups. However, as the AUC_1_ values of *α* and HFD are not sufficiently high we included the other features with AUC_1_ > 0.5 in building the model.

For the AI and HC + BP groups, the *α* has the highest AUC_2_ value of 0.76, while the AUC_2_ values of the other features fall between 0.5 and 0.6, thus unable to discriminate the AI and HC + BP cohort effectively. For the cases of CI and HC + BP, all features have their AUC_3_ values in the range of 0.5–0.6, which are too low for a single feature detection of the two groups. This can be also observed in the AUC_4_ values. We included all features in building the models.

### 3.2 Classification with machine learning algorithms

First, we consider the models that differentiate AI from CI. As shown on the flow chart of the model in Figure 3, we trained and tested four machine learning algorithms, namely, k-nearest neighbours (kNN), support vector machine (SVM), Naïve Bayes (NB) and random forests (RF), to determine the best model to classify AI and CI.

The main motivation of using machine learning is to prove our Hypothesis H1, namely that our automatic model can differentiate between AI and CI groups.

Two different sets of features were used to develop the models. The first feature set includes all the features, as shown in Table 3, 5 statistical, 4 dynamical and the actigraphy-derived sleep parameters TST, WASO and SWR. The latter were included to investigate their effect on the accuracy of the classification. The second set of features included 9 features, 5 statistical and 4 dynamical. The sleep parameters were excluded in order to reduce the effect that different methods of computation of these parameters from different devices can have on the classification. A significant benefit of this approach is that these characteristics are independent of the type of instrument that measures the signals and possible differences in sleep protocols. Five-fold cross-validation was used for validating each model. In addition, we executed the machine learning models five times and the average accuracy over 5 iterations was calculated for the two sets of features. The results are given in Table 4, left four columns for 12 features, right four columns for 9 features.

**TABLE 4:** Machine learning models using all features and 9 features (excluding sleeping parameters) to differentiate between AI v/s CI, AI v/s HC + BP, CI v/s HC + BP and Insomnia (AI + CI) v/s Healthy (HC + BP).

	12 features				9 features			
AI v/s CI								
Performance	kNN	SVM	NB	RF	kNN	SVM	NB	RF
Accuracy	0.79	**0.81**	0.78	0.78	0.79	**0.81**	0.79	0.78
Sensitivity	0.72	0.73	0.64	0.75	0.75	0.77	0.69	0.77
Specificity	0.85	0.89	0.91	0.80	0.83	0.86	0.88	0.78
Fscore	0.74	0.77	0.70	0.75	0.76	0.78	0.75	0.75
AUC	0.83	0.88	0.89	0.88	0.85	0.89	0.85	0.86
AI v/s HC + BP								
Accuracy	0.69	0.67	**0.72**	0.69	0.69	0.72	**0.74**	0.70
Sensitivity	0.45	0.31	0.37	0.44	0.46	0.51	0.42	0.47
Specificity	0.81	0.84	0.89	0.81	0.80	0.82	0.90	0.81
Fscore	0.46	0.34	0.45	0.48	0.46	0.51	0.50	0.48
AUC	0.66	0.70	0.77	0.72	0.67	0.80	0.79	0.74
CI v/s HC + BP								
Accuracy	0.63	**0.64**	0.62	0.58	0.59	**0.60**	0.63	0.56
Sensitivity	0.38	0.21	0.01	0.42	0.30	0.15	0.06	0.37
Specificity	0.77	0.88	0.98	0.68	0.76	0.87	0.96	0.67
Fscore	0.37	0.24	0.01	0.41	0.30	0.15	0.07	0.41
AUC	0.66	0.65	0.53	0.62	0.59	0.63	0.52	0.59
Insomnia (AI + CI) v/s Healthy (HC + BP)								
Accuracy	0.53	**0.62**	0.54	0.52	0.51	**0.57**	0.51	0.52
Sensitivity	0.43	0.62	0.51	0.52	0.44	0.62	0.48	0.51
Specificity	0.63	0.61	0.57	0.52	0.59	0.52	0.55	0.54
Fscore	0.54	0.60	0.48	0.51	0.53	0.51	0.50	0.51
AUC	0.55	0.65	0.53	0.54	0.53	0.60	0.52	0.56

The results with 9 features (Table 4, right part) show that the SVM model achieved the highest overall accuracy of 81%, with very well-balanced sensitivity of 77% and specificity of 86%. This was closely followed by the kNN, NB and RF. RF yielded 78% accuracy with balanced sensitivity and specificity. However NB and kNN obtained similar accuracy 79% in differentiating AI and CI groups, but kNN has a better-balanced sensitivity and specificity. The performance of the algorithms, which used all 12 features, is given in Table 4, left part. The SVM model remains the best-performing model. It accomplished 81% accuracy, well-balanced sensitivity of 73% and specificity of 89%. This was followed by kNN, RF and NB with the accuracy of 79%, 78% and 78%, respectively. NB performed worst with accuracy of 78%, sensitivity of 64%, and specificity of 91%. We conclude that the small reduction in performance and balance between accuracy, sensitivity and specificity is due to adding the sleep parameters as additional three features (accurately calculated for the CI group but approximated for the AI group).

**FIGURE 6:**
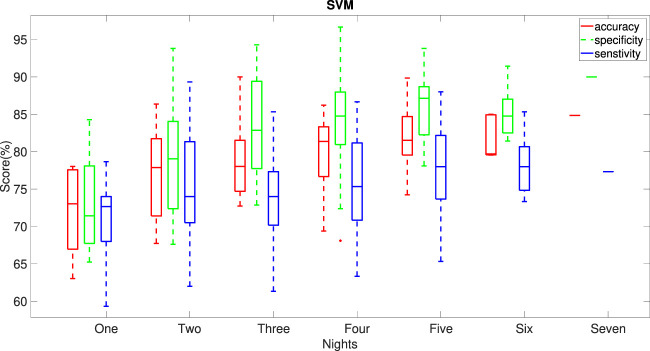
Dependence of the performance of the SVM model on the number of nights of actigraphy used in the model. Horizontal lines indicate median values for the performance metrics. All possible combinations of the given number of nights in the week of data were used to obtain the statistics.

We used the SVM model to calculate accuracy, specificity and sensitivity for all possible combinations of the given number of nights out of the seven nights of data. Figure 6 demonstrates that the performance of SVM model improves with increasing the number of nights included in the nocturnal actigraphy. Less than four nights of actigraphy leads to reduced performance. A minimum of 4 or 5 nights of actigraphy is required to achieve the median accuracy, sensitivity and specificity of at least 75%, with a minimum of 5 nights providing a more balanced performance. This further supports the results shown in our previous paper (Kusmakar et al., 2021).

The performance of the machine learning algorithms showed that the best performing model, SVM, can distinguish the AI group from the CI group, with a very high accuracy of 81% from averaging 7 nights of actigraphy. This model can classify the two groups with a relatively small number of parameters (9) extracted from the signal. This confirms our Hypothesis H1. Furthermore, we recommend SVM with 9 features for routine testing, as the statistical and dynamical features are objective measurements, extracted from the signal directly, and do not depend on sleep protocols. In order to investigate fully our Hypothesis H2, we have examined several designs of the model for classifications of other combinations of cohorts, namely AI v/s HC + BP, CI v/s HC + BP and insomnia (combined AI and CI) v/s HC + BP. We have performed the classifications with 9 and 12 features following similar cross-validation as in the previous case. Table 4 illustrates the results.

In the case of AI and healthy cohorts (HC + BP), the results show that the best-performing model, NB with 9 features, can differentiate AI from the healthy group with an accuracy 74%, closely matched by NB with 12 features, accuracy 72%, sensitivity 37% and specificity 89%.

Considering CI and the healthy cohorts, the best performing model, SVM with 12 features, has low accuracy 64% and unbalanced sensitivity and specificity, from averaging of seven nights of actigraphy. This shows that the CI group is less different from HC + BP compared to AI, which supports Hypothesis H2. Furthermore, when combining AI and CI in one insomnia group and comparing it to HC + BP, the SVM model can distinguish insomnia from the healthy group with a low accuracy of 62%. This confirms our previous results that the classification of insomnia from healthy sleep cannot be accurately done using averaging of actigraphic data over multiple nights as each night of sleep has to be classified separately. We have developed accurate models for such classification of insomnia and healthy sleep in our previous works (Angelova et al., 2020a; Kusmakar et al., 2021).

## 4 Discussion

Wrist-worn actigraphy devices allow for the non-obtrusive collection of activity data in a real-time environment. This paper presented data analysis and classification of multi-night physical activity data collected with two different actigraphy devices in two studies: acute insomnia study and healthy controls, and chronic insomnia study and healthy bed partners. We only included data from individuals from age 18 years–60 years in order to avoid the effect of ageing on sleep quality.

Data were cleaned and pre-processed and missing data were imputed. Healthy controls and bed partners were combined in one healthy cohort. After the cleaning and data transformations, we derived three groups of features from the actiwatch signal: statistical features, dynamical features and actigraphy-derived sleep parameters. The values of the features extracted from the signal, showed that the AI group has more physical activities possibly due to the stronger physical activity during the night compared to the CI group. This supports Hypothesis H1. Furthermore, the average values of the features shown in Table 3 indicate that the nocturnal physical activities of CI, while less than those of AI, are more similar to those of HC + BP. This may be due to adaptation to disturbed sleep for CI individuals. This supports Hypothesis H2.

The *p*-values and AUC values (AUC_1_) of the extracted features showed that for the AI and CI groups, except for two features, namely the complexity parameter *α* and Higuchi fractal dimension HFD, no other single feature is capable to differentiate between the AI and CI groups. However, their AUCs were not sufficiently high to make them reliable single markers for a diagnostic tool. For differentiating between AI and HC + BP group, AUC_2_ shows decreased importance of *α* and HFD. There are no significant prominent features for CI v/s HC + BP and insomnia v/s HC + BP groups as shown by AUC_3_ and AUC_4_ respectively (Table 3).

This required to develop machine learning algorithms in which two sets of features were submitted: the first set comprising 9 objective features, 5 statistical and 4 dynamical features, extracted directly from the signal, and the second set contained 12 features, where three sleep parameters, extracted from the signal, were added to the 9 statistical and dynamical features.

Four machine learning algorithms were deployed to incorporate two sets of features and classify the AI and CI groups. The algorithms selected were kNN, NB, RF and SVM. The ground truth for AI and CI groups, used in these machine learning models, was based on the clinical assessment of AI and CI individuals. The machine learning models were capable to effectively differentiate between acute and chronic insomnia.

The best-performing algorithm was SVM with an accuracy of 81% with 9 features (Table 4). SVM also demonstrated a very good balance of accuracy with sensitivity and specificity, and proved our Hypothesis H1, namely that we can distinguish acute insomnia from the chronic insomnia group using physical activity data only.

The performance of the best model SVM, as well as of all remaining models, was slightly reduced when the second set of features was used for the algorithms, in which three sleep parameters, TST, WASO and SWR, were added to the 9 features of the first set. This may be due to the accuracy of calculating the sleep parameters from the different recording devices.

We also noted that the differences in the sleep parameters for the AI study, compared with the corresponding parameters in the CI study (Table 3) may be due to the different protocols for staying in bed used in the two studies. The use of an older actiwatch (pre-2014), without the ability to detect lights out, also affected the accuracy of deriving the sleep parameters from the signal for the AI and HC + BP groups.

We investigated the classification of AI and the healthy group. The best-performing model was able to differentiate AI from healthy sleep with an accuracy of 74%. The classification of CI and HC + BP showed a subtle difference between CI and healthy sleep based on averaging over 7-night of the actigraphic data. This further supports Hypothesis H2, as CI individuals may have become more adjusted to sleeplessness compared to those with AI for which the changes resultant from the acute insomnia are still too raw and the organism and the respective homeostatic regulation have not adapted to these changes yet.

Furthermore, these results indicate that averaging over actigraphic data collected for 7 nights is not a suitable approach to differentiate insomnia from a healthy sleep, as individuals with insomnia may have good as well as bad nights of sleep and each night has to be classified separately. This was confirmed by considering all insomnia (AI + CI) v/s HC + BP groups, where the model achieved only 62% of accuracy (Table 4).

The purpose of this study was to distinguish AI from CI individuals based on the objective measurements from the physical movements, which was achieved with excellent accuracy. In addition, the machine learning models developed for other combinations of cohorts showed that the AI group has more prominent differences than the CI group when both were compared with the same HC + BP cohort.

## 5 Conclusion

Sleep normally is regulated, which means that the longer one stays awake, the longer and deeper one’s sleep will be. Health ailments, age, social and environmental factors affect the regularity, duration and quality of sleep. Sleep parameters such as total sleep time (TST), wake after sleep onset (WASO) and sleep-wake ratio (SWR) are explicitly different in healthy individuals and adults with insomnia. Adults with acute insomnia have less sleep time in comparison to adults with chronic insomnia (see Table 3). Frequent and long waking periods during the night can be observed in the night time actigraphy signals of AI and CI.

Not many studies have explored the area of homeostatic dis-regulation in regard to acute and chronic insomnia (Pigeon and Perlis, 2006). We analysed statistical and dynamical features and actigraphy-derived sleep parameters of 7 nights of actigraphy signals from two studies: acute insomnia individuals and healthy controls, and chronic insomnia individuals and their bed partners.

The extracted features showed that there are patterns of differences in the physical activities of the AI and CI group which supports our Hypothesis H2, namely that the observed changes in patterns for the CI and AI individuals may appear because the homeostasis drive has adjusted to sleeplessness in the individuals with CI, while for the individuals with AI, the changes are still too raw.

Our best-performing machine learning model, the SVM model, differentiated acute from chronic insomnia with an excellent accuracy of 81%, and balanced sensitivity and specificity. This proves our Hypothesis H1. Furthermore, the models also indicated that changes observed in acute insomnia were more prominent than those in chronic insomnia, when both were compared with the same healthy cohort. This further supports Hypothesis H2.

One limitation of this work is in the data collection, where two different protocols and different devices were used to measure physical movements. Another limitation is due to some imbalance in the age of the participants, where most of the HC + BP individuals are between the ages of 20 and 40, with other groups ranging up to 60. The reason is that the primary purposes of data collection in the AI and the CI studies were not for differentiation of AI from CI, and these data were re-used for the current work as secondary data.

At the same time, a clear advantage shown in this work is that with careful data pre-processing and feature extraction, it is possible to develop a machine-learning model capable to differentiate acute from chronic insomnia with high accuracy, sensitivity and specificity, based on data, collected with different wrist-worn actigraphy devices. This model represents a significant addition to our comprehensive suite of insomnia pre-screening and classification models, together with our previously developed models detecting insomnia from normal sleep (Angelova et al., 2020a; Kusmakar et al., 2021). The high degree of accuracy of the model makes it suitable for further development of a pre-screening tool for insomnia in a home setting.

## Data Availability

Publicly available datasets were analyzed in this study. This data can be found here: https://doi.org/10.5061/dryad.b8gtht7bh; https://royalsocietypublishing.org/doi/suppl/10.1098/rsif.2013.1112, Supplementary Material.
